# Neuroprotective effects of ketamine, lamotrigine, and ketoprofen combinations after pars plana vitrectomy and silicone oil injection

**DOI:** 10.1007/s10792-026-04256-8

**Published:** 2026-09-05

**Authors:** Mila W. Paques, Victor Bellanda, Renato Guizzo, Rubens C. Siqueira, Antônio R. M. Silva, Ingrid U. Scott, Wagner Ferreira dos Santos, Rodrigo Jorge

**Affiliations:** 1 Ophthalmology Division, Ribeirão Preto School of Medicine, University of São Paulo, Ribeirão Preto, Brazil; 2 Department of Biology, Faculty of Philosophy, Sciences and Literature, University of São Paulo, Ribeirão Preto, Brazil; 3 Cleveland Clinic Cole Eye Institute, Cleveland, OH USA; 4 Applied and Experimental Neurology Laboratory, Department of Neurology, Psychiatry and Medical Psychology, Ribeirão Preto School of Medicine, University of São Paulo, Ribeirão Preto, Brazil; 5 Departments of Ophthalmology and Public Health Sciences, Penn State College of Medicine, Hershey, PA USA

**Keywords:** Ketoprofen, Neuroprotection, Pars plana vitrectomy, Retinal ischemia, Silicone oil

## Abstract

**Purpose:**

This experimental study aims to assess the protective impact of combined treatment with ketamine (KTM), lamotrigine (LMT), and ketoprofen (KTP), agents that have demonstrated neuroprotective properties in other experimental contexts, on rabbit retinas in an experimental model of pars plana vitrectomy (PPV) and silicone oil injection (SOI).

**Methods:**

Thirty-six rabbits were divided into six treatment groups: saline, KTP, KTM+LMT, KTM+KTP, LMT+KTP, and KTM+LMT+KTP. All rabbits underwent PPV and SOI in the right eye. The left eyes served as negative controls. Histological assessments were conducted to evaluate cell densities and structural changes in the ganglion cell layer (GCL), the inner nuclear layer (INL), and the outer nuclear layer (ONL). Apoptosis was evaluated using the TUNEL method.

**Results:**

The KTP group exhibited reduced apoptosis and partial protection in all retinal layers. The KTM+LMT combination significantly protected the GCL. The KTM+KTP group showed notable protection in the INL. The LMT+KTP combination was effective for both the INL and GCL. The KTM+LMT+KTP combination provided the most comprehensive protection across the ONL and INL. The cell densities in the ONL, INL, and GCL were significantly greater in the treatment groups than in the positive control group.

**Conclusion:**

The combination of KTM, LMT, and KTP exhibited significant neuroprotective effects on rabbit retinas following PPV and SOI. These findings suggest the potential of this combination treatment as a therapeutic strategy for retinal pathologies.

## Background

Pars plana vitrectomy (PPV) with silicone oil injection (SOI) is widely used in the management of complex retinal detachments and other severe vitreoretinal disorders requiring long-term internal tamponade.[1] While silicone oil is effective in maintaining retinal attachment, several studies have reported unexplained postoperative visual loss and progressive retinal degeneration in a subset of patients following SOI, even in the absence of recurrent retinal detachment or other identifiable structural complications, with incidence rates ranging from 3% to nearly 30% depending on patient selection and duration of tamponade [1, 2]. These findings suggest that silicone oil may induce direct or indirect neurotoxic effects on retinal tissue [3].

Experimental and clinical studies suggest that silicone oil-associated retinal damage may result from a combination of mechanical compression, impaired retinal perfusion, and biochemical toxicity, ultimately leading to retinal ischemia and neuronal degeneration [4]. Experimental and clinical studies have demonstrated that prolonged silicone oil retinal tamponade can lead to selective structural damage within the retina, including thinning or complete disappearance of the outer plexiform layer (OPL) and alterations in inner retinal layers [5–9]. Retinal ischemia and metabolic stress may trigger the production of reactive oxygen species, and excitotoxic signaling pathways that ultimately lead to neuronal death and visual dysfunction [4, 10, 11]. The biochemical factors of silicone oil toxicity result from its composition, which includes linear and cyclic low molecular weight components that can spread to other ocular tissues, leading to long-term ocular damage [5, 12, 13].

Retinal histological changes, including the development of vacuoles in the external photoreceptor segments, thinning or disappearance of the OPL, and a significant reduction in the cell processes of horizontal and bipolar cells, as well as the synaptic terminals of photoreceptors, have been documented following experimental PPV and SOI [5, 14, 15]. Furthermore, there was a decrease in the thickness of the photoreceptor layer, nerve fiber layer edema, intercellular edema affecting bipolar and photoreceptor cells, and hydropic vacuolization of ganglion cells [5, 16, 17]. These changes, along with the associated clinical findings of chronic progressive visual deterioration following the procedure, suggest that PPV with SOI may produce a cascade of ischemic and excitotoxic injury affecting multiple retinal layers, particularly synaptic and neuronal structures within the OPL, inner nuclear layer (INL), and ganglion cell layer (GCL), which are highly susceptible to metabolic stress [18–20].

Strategies aimed at neuroprotection may help mitigate retinal damage following silicone oil tamponade. The objective of neuroprotection is to prevent or slow the progression of diseases by inhibiting neuronal death [21]. The retina contains a diverse array of neurotransmitters, including L-glutamate, an excitatory amino acid vital for mediating ischemic damage through the overactivation of N-methyl-D-aspartate (NMDA) channels [11, 22–24]. Several studies have investigated the effectiveness of various neuroprotective strategies, such as NMDA receptor antagonists, antiepileptic medications, and anti-inflammatory agents, in a range of experimental models [25–29]. Among these agents, ketamine, lamotrigine, and ketoprofen have shown potential neuroprotective properties.

Ketamine (KTM) is a noncompetitive antagonist of NMDA receptors. It has shown neuroprotective effects in various neurological conditions, including ischemic stroke and traumatic brain injury [30]. KTM has been tested intramuscularly for neuroprotection after PPV with SOI[26] and intravenously for neuroprotection in a rabbit model of glaucoma [28]. Additionally, it has been tested in an intravitreal form for neuroprotection after ischemia and reperfusion in rabbit eyes, demonstrating increased retinal neuronal survival, primarily in the inner nuclear layer (INL) and ganglion cell layer (GCL) when compared to that of operated animals receiving placebo [26, 31].

Similarly, lamotrigine (LMT), an antiepileptic drug, has exhibited neuroprotective effects through its ability to inhibit glutamate release and reduce neuronal excitotoxicity [27, 29]. LMT acts by blocking pre- and postsynaptic voltage-dependent sodium channels and postsynaptic voltage-dependent calcium channels, thus stabilizing neuronal membranes and reducing the release of excitatory neurotransmitters, particularly L-glutamate and aspartate [32]. In a previous study, oral lamotrigine provided a protective effect against the adverse consequences of PPV with SOI on rabbit eyes. Specifically, it prevented retinal cell apoptosis and minimized glial cell activation [27].

Ketoprofen (KTP), a nonsteroidal anti-inflammatory drug (NSAID), has been widely used for its anti-inflammatory and analgesic effects. Some studies suggest that it may also possess neuroprotective properties by modulating inflammatory pathways and reducing oxidative stress [33–35]. KTP inhibits prostaglandin synthesis by inactivating cyclooxygenase, an enzyme that catalyzes the formation of prostaglandin precursors from arachidonic acid. In addition to directly halting inflammation in the brain tissue, the reduction in prostaglandin biosynthesis prevents an increase in vascular permeability and in the migration of polymorphonuclear leukocytes [36].

Given the potential effects of these agents, we conducted an experiment to evaluate the synergistic impact of their combinations in a rabbit model of PPV with SOI. We aimed to determine whether these compounds could protect against retinal ischemia and oxidative stress, ultimately preserving retinal cell count and general histology. KTM and LMT alone were not tested in this study since they had previously been investigated in other studies conducted by our group [26, 27].

## Methods

Thirty-six female New Zealand rabbits weighing between 2.0 and 2.5 kg were used. Each rabbit was housed separately in individual Plexiglas cages and provided with unrestricted access to water and food. The rabbits were subjected to a 12-h light/dark cycle for 48 h before commencing the experiment to establish a consistent circadian rhythm, and the temperature in the facility was maintained between 23 °C and 25 °C. The rabbits were handled according to the Brazilian Society for Neuroscience and Behavior guidelines, in compliance with Federal Law N. 11,794 (August 10, 2008) and the ARVO Statement for the Use of Animals in Ophthalmic and Vision Research.

### Drug administration

The 36 rabbits were equally divided into six groups. The first group was administered daily intramuscular injections of placebo solution (saline, 150 mM in 0.5 ml). The second group received daily intramuscular injections of KTP (10 mg/kg in 0.5 mL). The third group was administered daily oral injections of KTM (50 mg/kg) combined with LMT (25 mg/kg), each in a volume of 0.5 mL. The fourth group received daily oral injections of KTM (50 mg/kg) and KTP (10 mg/kg), both in a volume of 0.5 mL. The fifth group was administered daily oral injections of LMT (25 mg/kg) and KTP (10 mg/kg), each in a volume of 0.5 mL. The sixth group received daily oral injections of KTM (50 mg/kg), LMT (25 mg/kg), and KTP (10 mg/kg), each in a volume of 0.5 mL. This regimen was followed for four weeks in all groups.

### Pars plana vitrectomy

Each rabbit was initially sedated with a 2 mL intravenous dose of sodium thiopental (50 mg/kg), with further doses administered as necessary. Dilation of the pupils was achieved using a combination of 1% tropicamide and 2.5% phenylephrine hydrochloride.

The right eyes were subjected to a standard three-port PPV, using the DORC Harmony Total vitrectomy machine (DORC, Netherlands). Sclerotomies were performed 1 mm posterior to the limbus. Post-PPV, the vitreous cavity of the right eye of each rabbit was filled with 1 mL of 5000-centistoke silicone oil (Ophthalmos, Brazil). The left eyes served as unoperated controls.

### Histological evaluation

Four weeks after the operative interventions and without removal of the silicone oil, the rabbits were euthanized with sodium thiopental. The ocular globes were then submerged in a preservative mixture comprising 85% ethanol, 10% formaldehyde, and 5% acetic acid for one day. To ensure proper positioning, a linear incision was made in the superonasal quadrant of the sclera of each eye, and the ocular components were separately excised. The eye globes were subsequently halved parallel to the optic nerve, below the horizontal meridian. The lower halves were excluded from further examination, whereas the upper halves underwent dehydration in ethanol solutions ranging from 70 to 100% before being embedded in paraffin. Retinal tissue cross-sections. (5 μm thick) were prepared and subjected to hematoxylin and eosin (H&E) staining. The sections were then examined under a Zeiss Axiophot microscope (Zeiss, Germany). For uniformity, the retinal sections chosen for qualitative assessment were all sourced from an identical region (1 mm superior to the optic disc) across all eyes. The evaluations focused on identifying qualitative alterations such as intercellular edema, disruption of cellular architecture, and vacuolation within the cytoplasm.

To evaluate apoptosis via the terminal deoxynucleotidyl transferase dUTP nick end labeling (TUNEL) technique [37], retinal sections were deparaffinized in xylene followed by rehydration through a series of ethanol dilutions (100–50%). After rinsing in phosphate-buffered saline (PBS), the slides were laid flat and exposed to 20 μg/mL proteinase K (Promega, Madison, WI, USA) for 10 min, rinsed again in PBS, and covered with 100 μL of equilibration buffer (Promega) for an additional 10 min. Each section was then treated with a mixture containing 45 μL of equilibration buffer, 5 μL of nucleotide mix (12-deoxyuridine triphosphate–fluorescein), and TdT enzyme (Promega) and incubated at 37 °C for one hour in a moisture-controlled chamber. The reaction was halted by immersing the slides in 2X SSC (Promega) followed by a PBS wash; the slides were then sealed with Fluoromount–G (EMS, Hatfield, PA, USA) and glass coverslips. Evaluation was conducted under a fluorescence microscope (Eclipse E800, Nikon, Melville, NY, USA) equipped with a fluorescein isothiocyanate filter (excitation wavelength: 488 nm; emission wavelength: 525 nm) and a 40× objective lens. All images were captured at a consistent magnification and exposure time on the same day using a digital camera (DXM 1200, Nikon, Japan) and Nikon ACT-1 software.

### Quantitative analyses

Light microscopy (Axiophot, Zeiss, Germany) was utilized to examine and photograph five microscopic fields (equivalent to 160 × 474 pixels each) from a single transverse section located 1 mm above the optic nerve in the superior retinal region of each eye. The sample included six groups of eyes-saline-operated, KTP-operated, KTM-LMT-operated, KTP-KTM-operated, KTP-LMT-operated, and KTM-LMT-KTP-operated-with six eyes per group. Additionally, 36 images were obtained from the left eyes, which served as the negative control group. Cell counts (expressed as the mean densities in cells/mm^2^ ± standard error of the mean [SEM]) were quantified using the KS 400 software (Zeiss, Germany). Statistical analysis was performed using one-way analysis of variance (ANOVA) followed by the Student–Newman–Keuls post-hoc test. A *p*-value < 0.05 was considered to indicate statistical significance.

## Results

**Fig. 1:**
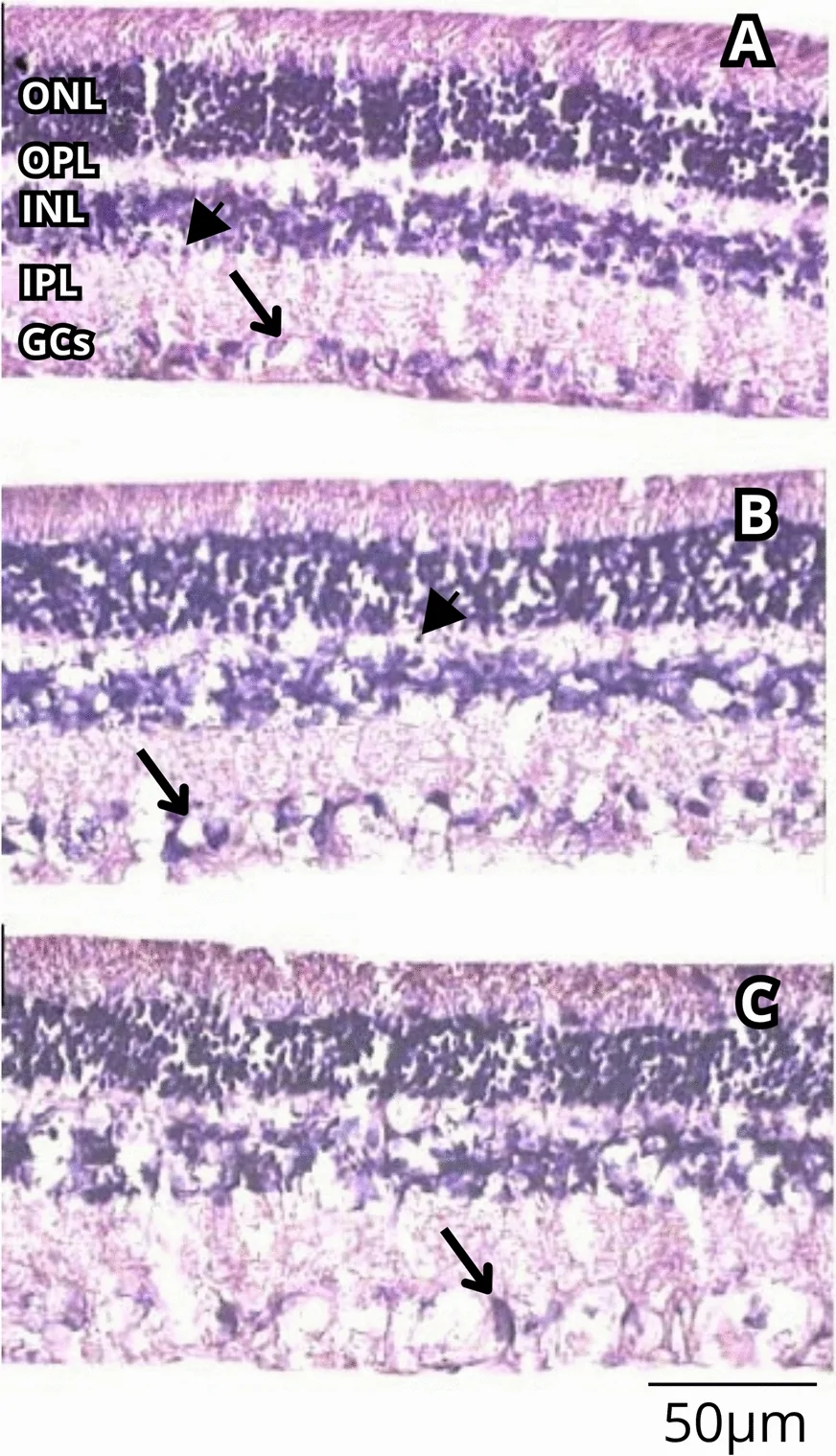
Histological comparison of retinas from the negative and positive control groups. In **A**, a negative control (left eye, unoperated) retina displays preserved retinal cell layers and maintained architecture, with rare pyknotic nuclei (arrowhead) and pericellular edema (arrow). In contrast, **B**, a positive control (saline-operated) retina revealed decreased cell counts in the GC layer, INL, and ONL, with more pyknotic nuclei (arrowhead) in the OPL and significant pericellular edema around GCs. Additionally, there are intracellular edema and structural disarray in the INL and ONL, cytoplasmic vacuolization in the GCs (arrow), and intracellular edema and matrix disorganization within the IPL. The retinas of animals in the KTP-operated group **C** exhibited cell disorganization, matrix disarray in the OPL and IPL, and cytoplasmic vacuolization in the GCs (arrow). Hematoxylin–eosin. *Abbreviations: ONL* = *Outer Nuclear Layer; OPL* = *Outer Plexiform Layer; INL* = *Inner Nuclear Layer; IPL* = *Inner Plexiform Layer; GCs* = *Ganglion Cells*

**Fig. 2:**
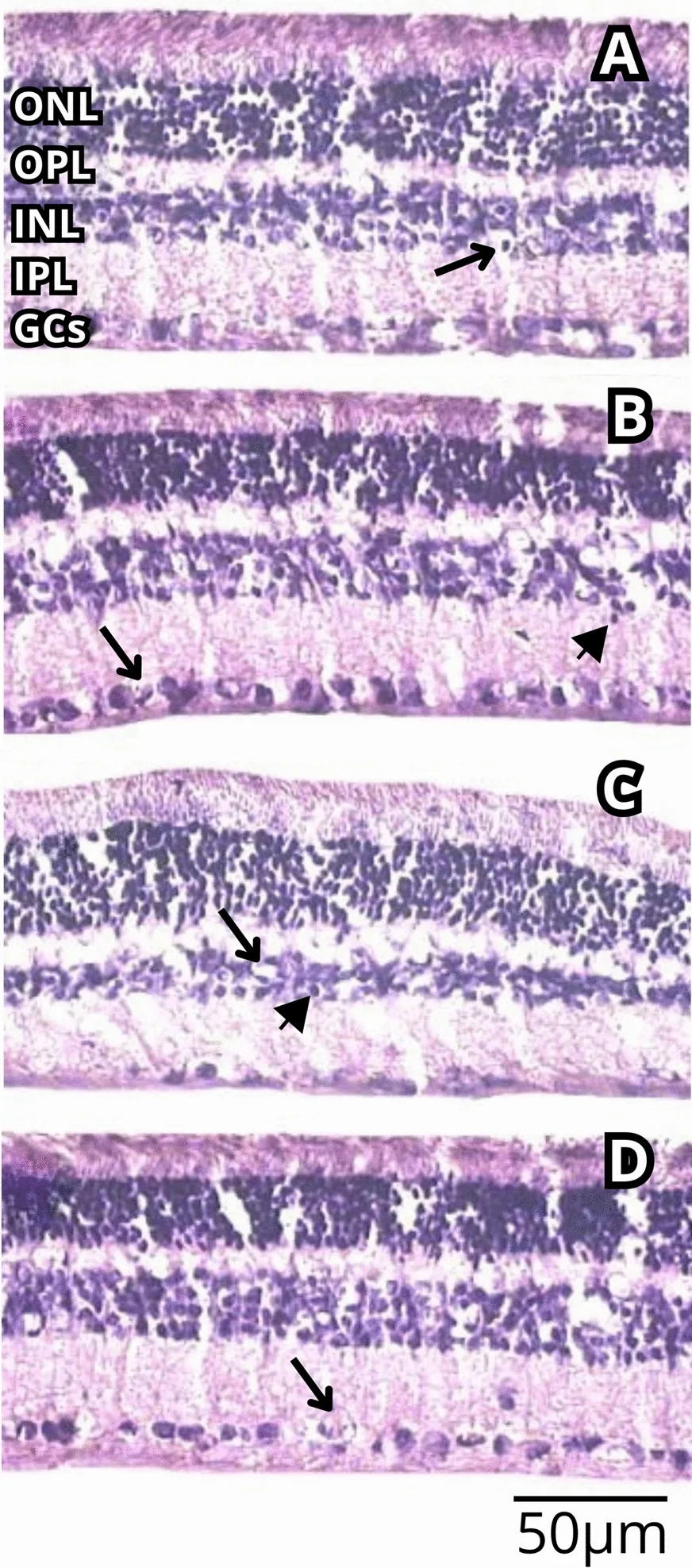
Histological analysis of **A** KTM-LMT-operated retinas, **B** KTM-KTP-operated retinas, **C** LMT-KTP-operated retinas, and **D** KTM-LMT-KTP-operated retinas. Overall retinal lamination was preserved across all groups. Occasional focal alterations were observed, including small vacuoles within the INL and GCs (arrows) and rare pyknotic or displaced nuclei within the INL (arrowheads). These findings were infrequent and comparable to those observed in negative control specimens. Hematoxylin–eosin. *Abbreviations: ONL* = *Outer Nuclear Layer; OPL* = *Outer Plexiform Layer; INL* = *Inner Nuclear Layer; IPL* = *Inner Plexiform Layer; GCs* = *Ganglion Cells*

Figures 1 and 2 illustrate the histopathological examination of the retinas in each operated group and the negative control group (unoperated, no drugs administered). The control group, consisting of 36 unoperated eyes (six from each group), displayed no histological signs of intracellular edema or cellular disorganization in the retinas (Fig. 1-A). The average cell density, expressed as cells/mm^2^ ± SEM, was 470 ± 28 in the outer nuclear layer (ONL), 95 ± 7 in the INL, and 15 ± 2 in the GCL. No significant differences were observed in the cell counts of the ONL, INL, or GCL among the 36 control retinas.

In contrast, the retinas of rabbits in the saline-operated group (positive control) showed signs of intracellular edema, cellular disorganization, reduced cell counts, and thinning in the INL; extracellular edema and matrix disorganization in the OPL and IPL; and vacuolization of the cytoplasm in the ganglion cells (Fig. 1B). These structural alterations are consistent with previously described retinal injury following SOI, which could potentially be attributed to ischemia-induced excitotoxicity, oxidative injury, or mechanical stress on retinal tissue [5, 9, 38, 39]. Similarly, the retinas of the KTP-operated group exhibited cellular disorganization, matrix disorganization in the OPL and IPL, and cytoplasmic vacuolization in the ganglion cells. These alterations were not observed in the retinas of rabbits in the KTM-LMT-, KTM-KTP-, LMT-KTP-, or KTM-LMT-KTP-operated groups (Fig. 2).

**Fig. 3:**
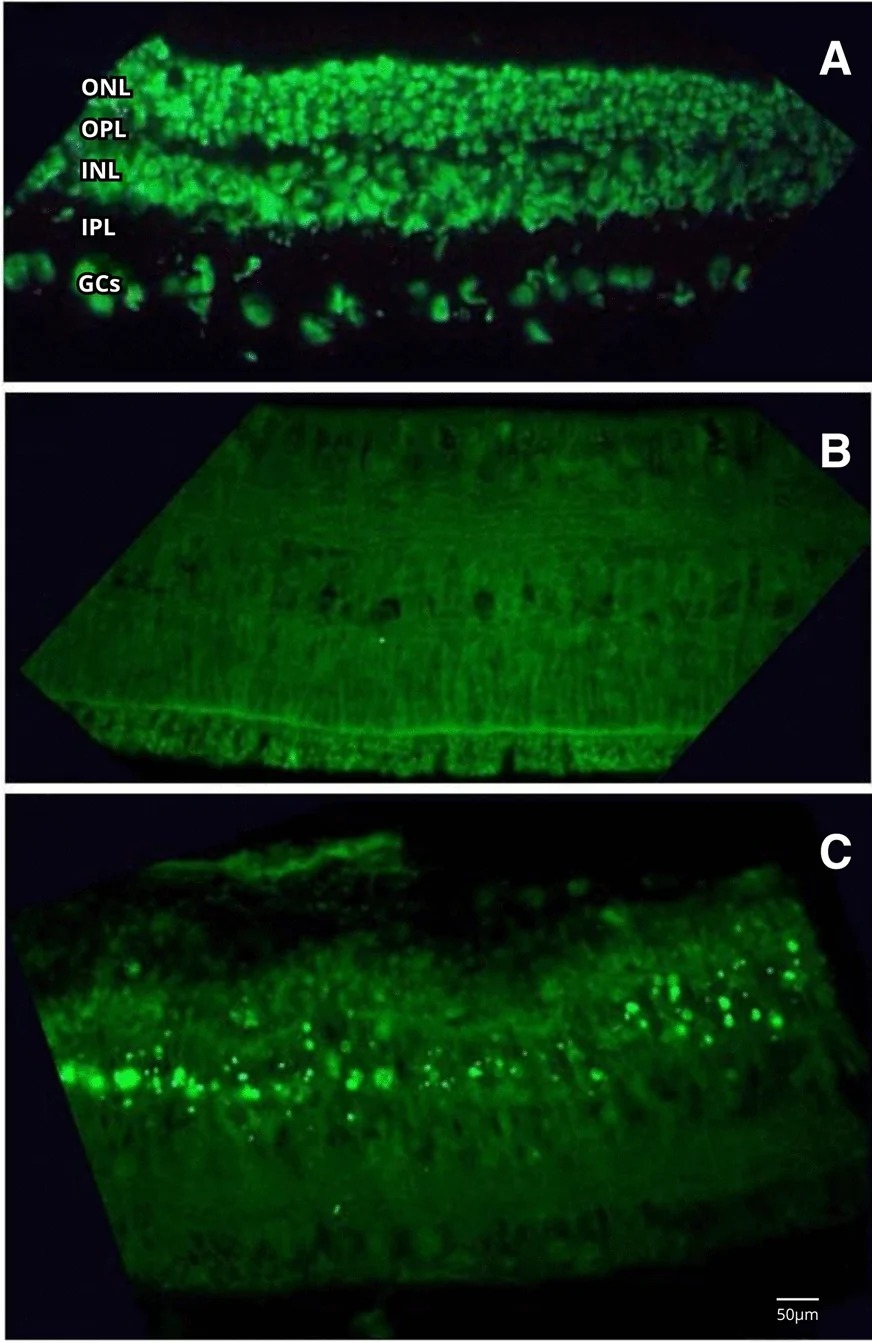
Detection of apoptosis in rabbit retinas using the TUNEL method. **A** The positive control retina post-PPV and SOI showed increased apoptosis, as indicated by the bright fluorescent signals. **B** Negative control retina from unoperated eyes demonstrates minimal fluorescence, suggesting low levels of apoptosis. **C** KTP-operated retinas exhibited intermediate levels of apoptosis between those of the positive and negative controls. *Abbreviations: ONL* = *Outer Nuclear Layer; OPL* = *Outer Plexiform Layer; INL* = *Inner Nuclear Layer; IPL* = *Inner Plexiform Layer; GCs* = *Ganglion Cells*

**Fig. 4:**
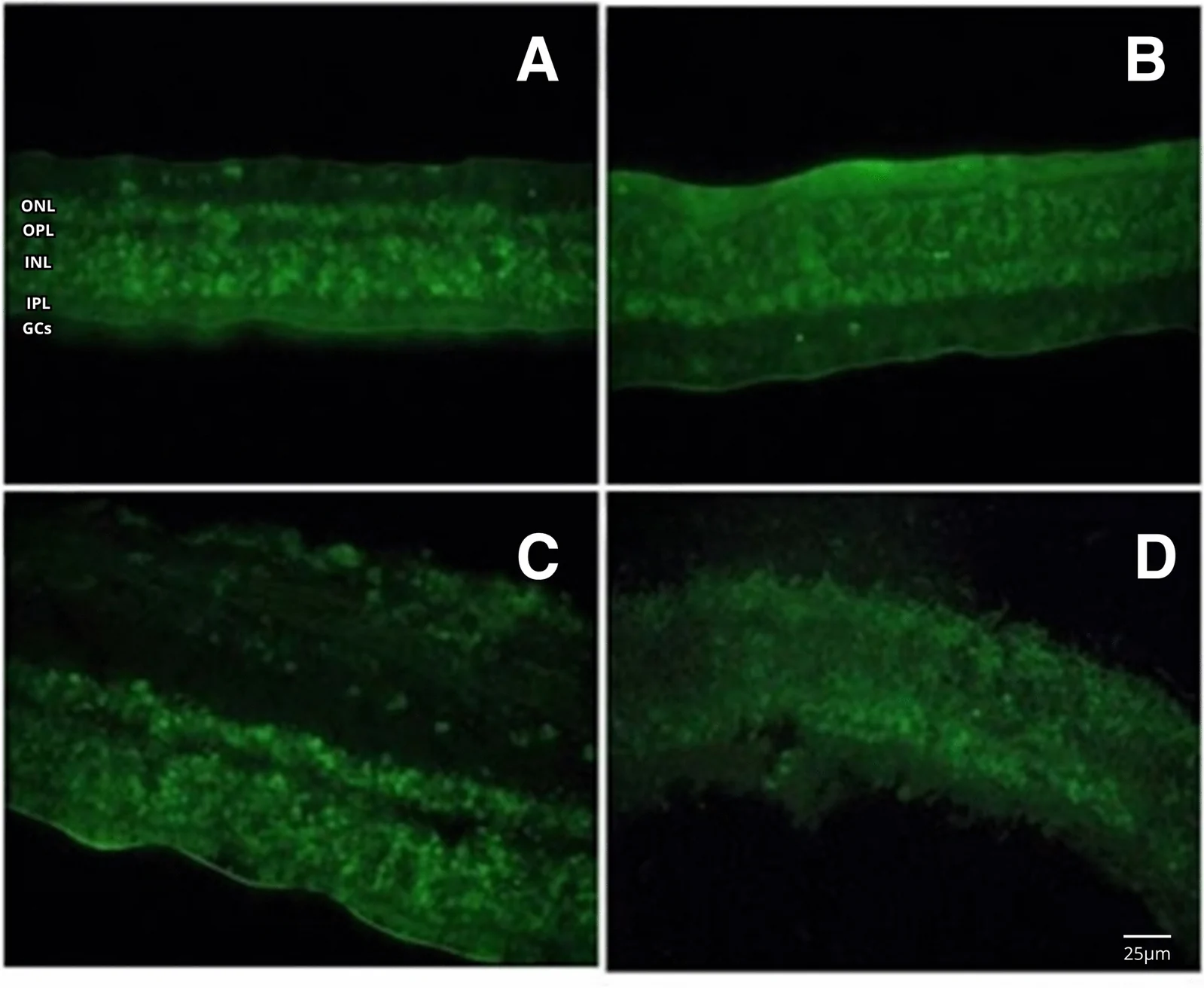
Detection of apoptosis via TUNEL staining in **A** KTM-LMT-operated retinas, **B** KTM-KTP-operated retinas, **C** LMT-KTP-operated retinas, and **D** KTM-LMT-KTP-operated retinas. These groups showed minimal fluorescence, suggesting low levels of apoptosis, similar to the observations made in negative control eyes, suggesting that the interventions led to apoptosis levels akin to the baseline observed in unoperated retinas. *Abbreviations: ONL* = *Outer Nuclear Layer; OPL* = *Outer Plexiform Layer; INL* = *Inner Nuclear Layer; IPL* = *Inner Plexiform Layer; GCs* = *Ganglion Cells*

Figures 3 and 4 demonstrate the quantification of apoptosis in every operated group, as well as in one positive (saline-operated) and one negative (unoperated, no drugs administered) control groups. The positive control assessed the test’s reliability. In KTP-operated retinas, we detected fluorescent spots similar to those in the positive control group, indicating the occurrence of apoptosis in this group. However, their intensity was not as pronounced as in the positive control, suggesting a potential attenuation of the effect in KTP-operated retinas.

In the ONL, the cell densities in the positive control (saline-operated) retinas were significantly lower than those in the negative control (unoperated) retinas. However, the cell densities in the KTP-operated and KTM-LMT-KTP-operated retinas were greater than those in the saline-operated samples. The mean cell counts were 5248 ± 339 in saline-operated eyes, 11,657 ± 1466 in KTP-operated eyes, 10,449 ± 1441 in KTM-LMT-operated eyes, 9613 ± 998 in KTM-KTP-operated eyes, 9834 ± 1319 in LMT-KTP-operated eyes, and 11,377 ± 266 in KTM-LMT-KTP-operated eyes (Table 1). Thus, KTP was responsible for 82%, KTM-LMT for 73%, KTM-KTP for 67%, LMT-KTP for 69%, and KTM-LMT-KTP for 80% protection (Table 1). In the ONL, there was no significant difference in the cell counts between any of the nonplacebo treatment groups.

**Table 1:** - Cell densities in the outer nuclear layer (ONL), inner nuclear layer (INL) and ganglion cell layer (GCL), respectively. * Indicates significant difference (*p*<0.05) compared to contralateral negative control retinas. ** indicates significant difference compared to positive control retinas (unpaired Student’s *t*-tests and One Way Analysis of Variance using Student–Newman–Keuls post-test method)

Groups	ONL	INL	GCL
Negative control (left eyes)	14,245 ± 850	3766 ± 266	562 ± 62
Positive control	5,248 ± 339*	1568 ± 93*	149 ± 26*
KTP	11,657 ± 1466**	3406 ± 522**	277 ± 35
KTM-LMT	10,449 ± 1441	3370 ± 895	538 ± 202**
KTM-KTP	9613 ± 998	2524 ± 76**	221 ± 31
LMT-KTP	9834 ± 1319	3425 ± 529**	491 ± 108**
KTM-LMT-KTP	11,377 ± 266**	3738 ± 285**	272 ± 57

In the INL, the cell densities in the positive control (saline-operated) retinas were significantly lower than those in the negative control (unoperated) retinas. Conversely, the cell densities in the KTP-operated, KTM-KTP-operated, LMT-KTP-operated, and KTM-LMT-KTP-operated retinas were significantly greater than those in the saline-operated retinas. The mean cell counts were 1568 ± 93 in saline-operated eyes, 3406 ± 522 in KTP-operated eyes, 3370 ± 895 in KTM-LMT-operated eyes, 2524 ± 76 in KTM-KTP-operated eyes, 3425 ± 529 in LMT-KTP-operated eyes, and 3738 ± 285 in KTM-LMT-KTP-operated eyes (Table 1). Therefore, in the INL, KTP was responsible for 90%, KTM-LMT for 89%, KTM-KTP for 67%, LMT-KTP for 91%, and KTM-LMT-KTP for 99% protection. There was no statistically significant difference in INL cell counts among the retinas of any of the nonplacebo treatment groups.

Regarding the GCL, cell densities in the positive control (saline-operated) and KTP-operated retinas were significantly lower than those in the negative control (unoperated) retinas. However, the cell densities in the LMT-KTP- and KTM-LMT-operated eyes were markedly greater than those in the saline-operated eyes. The mean cell count was 149 ± 26 in saline-operated eyes, 277 ± 35 in KTP-operated eyes, 538 ± 202 in KTM-LMT-operated eyes, 221 ± 31 in KTM-KTP-operated eyes, 491 ± 108 in LMT-KTP-operated eyes, and 272 ± 57 in KTM-LMT-KTP-operated eyes (Table 1). Thus, KTP was responsible for 49%, KTM-LMT for 96%, KTM-KTP for 39%, LMT-KTP for 87%, and KTM-LMT-KTP for 48% protection. There was no statistically significant difference in cell counts in the GCL among the retinas of any of the nonplacebo treatment groups.

No significant differences in IOP among the groups were present before, during, or after the interventions, and no significant IOP changes were observed in any group during the study.

## Discussion

The results demonstrated that different combinations of KTM, LMT, and KTP had neuroprotective effects on rabbit retinas after PPV and SOI. The combinations were associated with increased retinal neuronal survival in all investigated layers (ONL, INL, and GCL), corroborating with findings from other studies conducted by our group that tested the use of KTM and LMT alone [26, 27, 31].

The retinal histological findings after PPV and SOI, such as inter- and intracellular edema in the ONL and INL, as well as ganglion cell degeneration and vacuolization, have also been described in previous studies [5, 16, 17]. These alterations typically emerge approximately 30 days postprocedure due to two factors. First, mechanical vitrectomy exposes the retina directly to silicone oil, unlike gas compression and corneal incision vitrectomy, where the remaining vitreous cortex protects the retina, delaying histological changes [13, 40]. Second, the fluid-gas exchange during surgery ensures the complete removal of vitreous cavity fluid and thorough silicone oil filling, intensifying the mechanical impact on the superior retina [5].

The toxicity of silicone oil involves both mechanical and biochemical pathways that can ultimately lead to retinal ischemia, excitotoxic neuronal injury, and disruption of retinal cellular organization [4, 9–11, 38]. The mechanical impact is illustrated by the disappearance of the OPL in the upper retina, as observed by Doi and Refojo (1994) [5]. In our study, we observed a significant reduction in GCL thickness in rabbit retinas treated with silicone oil, compared to control retinas. This difference could be due to the shorter exposure time to silicone oil in our experiment (1 month) compared to that in the study by Doi and Refojo (3–6 months). Extended mechanical pressure from silicone oil on the upper retina can induce ischemia over time, leading to the release of vesicular glutamate into the extracellular space [4, 11, 41]. Excessive glutamate levels cause overstimulation of NMDA receptors, triggering the opening of specific channels for sodium and calcium ion influx. This ion influx, along with the subsequent water influx, could explain the intracellular edema observed in saline-operated retinas. These ionic disturbances can disrupt cytoskeletal integrity and synaptic organization within retinal layers, contributing to the cellular disorganization [11, 22–24]. Additionally, silicone oil is composed of low molecular weight elements, both linear and cyclic, that can permeate other ocular tissues, contributing to cellular toxicity [42]. Thus, the increase in glutamate levels contributes to the disruption of ion balance during ischemic-hypoxic neuronal injury, promoting the influx of sodium and calcium ions through channels associated with NMDA receptors [22, 25, 28].

The neuroprotective effects of ketamine can be attributed to its ability to block calcium channels associated with NMDA receptors, thereby inhibiting ion influx and subsequent cell death [31]. Several studies have shown the effects of ketamine and its derivative, esketamine, on various models of neuronal injury and retinal damage [26, 28, 30, 31, 43]. Tang et al. [30] demonstrated that esketamine effectively reduces oxidative stress, neuronal damage, and cell death in the cortex in traumatic brain injury models. They found that esketamine enhances autophagy and alleviates oxidative stress through AMPK/mTOR-dependent TFEB nuclear translocation-induced autophagy and the TFEB/Nrf2-induced antioxidant system. Similarly, Tsukahara et al. [28] reported that ketamine may alleviate ischemic injury by antagonizing NMDA receptor-mediated toxicity.

Arango-Gonzalez et al. [43] reported that pretreatment with ketamine protects retinas against light-induced damage and reduces photoreceptor cell death. They suggested that its neuroprotective effects are associated with increased neuronal and astroglial viability, preservation of neuronal morphology, inhibition of NF-κB activity, and potential activation of nitric oxide synthase. Furthermore, ketamine has been implicated in other mechanisms, such as inhibition of rhodopsin regeneration, modulation of mitochondrial function, and preservation of cellular energy status [43].

In a previous study conducted by our group, we explored the protective effects of intramuscular ketamine after inducing retinal ischemia using a saline pressure column [11]. Our qualitative assessment revealed a specific preservation of neurons in the GCL. Nonetheless, systemic administration of ketamine can result in adverse effects, including psychosis, sleepiness, sleeplessness, and modifications in sensory experiences, such as alterations in the perception of taste, cold, and heat [44]. To address this issue, an alternative administration route, intravitreal, was proposed by Dourado et al.[31] Initially, the investigators determined the safe dosages of intravitreal ketamine using a hen embryo model. Subsequently, they compared the effects of intravitreal ketamine and saline in rabbit eyes after ischemic injury and reperfusion. In line with our observations, histological examination revealed that the retinas of eyes subjected to saline operation displayed swelling and cellular disarray in the ONL and INL, diminished cellular density in the ONL, INL, and GCL, as well as cytoplasmic vacuoles in ganglion cells. Conversely, the retinal layers of eyes treated with ketamine were histologically comparable to those of the saline-control and ketamine-control eyes.

Lamotrigine binds to and inhibits voltage-gated sodium channels, stabilizing neuronal membranes and reducing the release of glutamate and aspartate. This inhibition of sodium influx decreases action potentials and prevents excessive intracellular sodium buildup. By preserving the sodium gradient, lamotrigine enables the sodium glutamate transporters to efficiently reuptake glutamate, effectively terminating glutamate neurotransmission [27, 29].

Sandalon et al. [29] investigated the effects of lamotrigine on acute and chronic ocular hypertension models in rats. The researchers administered different doses of lamotrigine, evaluated retinal function using ERG, measured retinal ganglion cell and axon densities, and assessed inflammatory reactions. They observed a significant decrease in b-wave amplitudes, ranging from 40 to 66%, in both treatment groups six days after IOP elevation. Importantly, there was no significant difference between the treatment groups. However, the study revealed a noteworthy finding: lamotrigine-treated rats exhibited a partial recovery of b-wave amplitudes between days 6 and 14 following the procedure, indicating a potential beneficial effect.

Lamotrigine also shows some promise for neuroprotection in optic neuritis [45]. Experimental evidence suggests that partial blockade of sodium channels can protect against axonal loss and improve clinical outcomes in multiple sclerosis (MS) models [46]. The so-called "sodium hypothesis" suggests that axonal injury arises from excessive energy demands due to increased sodium loading and subsequent calcium ion accumulation, triggering degradative enzyme activity [45]. The inhibition of mitochondrial respiration by inflammatory mediators such as NO further contributes to axonal damage. Evidence supports the neuroprotective effects of partial sodium channel blockade in both normal optic nerve axons and experimentally demyelinated axons exposed to NO [47]. Furthermore, the presence of Nav 1.6 sodium channels in macrophages and microglia within acute MS lesions, with their upregulation upon activation, suggests a mechanism for axonal injury through CD4+ T-cell proliferation, inflammatory cytokine production, and NO release, suggesting that sodium channel blockade is a potential neuroprotective strategy in acute optic neuritis [48].

In the present study, we observed that the combination of lamotrigine with either ketamine or ketoprofen significantly protected the INL and GCL. Furthermore, when lamotrigine was combined with both drugs, it exhibited protection in both the ONL and INL. These findings are consistent with our previous research[27], where we investigated the isolated effects of lamotrigine, and align with the observations made by Smith and Meldrum [49] and Calabresi et al. [50], who reported its protective effects in rat models of brain ischemia. Collectively, these data further support the neuroprotective potential of lamotrigine, whether used alone or in combination with other agents.

The potential neuroprotective effects of ketoprofen, an NSAID, are relatively understudied. However, some studies suggest that it may have neuroprotective properties by modulating inflammatory pathways and reducing oxidative stress [33–35]. Ketoprofen inhibits prostaglandin synthesis by inactivating cyclooxygenase, thereby reducing vascular permeability and leukocyte migration [36]. Marconcini et al. (1993) reported a reduction in retinal edema in patients treated with nimesulide, another NSAID, after laser treatment of ocular diseases [51].

In the present study, isolated ketoprofen treatment yielded less favorable results than the combination treatments. Histological analysis revealed cell disorganization, matrix disorganization in the OPL and IPL, cytoplasmic vacuolization in ganglion cells, and apoptosis in the retinas of rabbits treated with ketoprofen alone. However, despite these negative effects, the KTP-operated group still exhibited significant improvements in cell density in the ONL, INL, and GCL compared with the saline-operated group. KTP provided 82% protection in the ONL, 90% protection in the INL, and 49% protection in the GCL. Interestingly, the combination groups involving KTP with LMT and KTM showed even greater levels of protection. These findings suggest the potential synergistic effects of combination treatments and highlight the need for further studies to assess the effects of NSAIDs, including ketoprofen, on neuroprotection after retinal stress.

Although this study provides valuable insights into the neuroprotective effects of these drug combinations, it is important to consider its potential limitations. First, the study was conducted using rabbit models, and extrapolation of the findings to human patients may be limited. The absence of a macular region in the rabbit retina precluded the evaluation of parafoveal GCL, which is a key structure in human central vision and a relevant target in the context of silicone-oil-associated visual loss [52]. Rabbits are afoveate animals; instead of a fovea, their retina possesses an “area centralis” or, more specifically, a horizontally oriented visual streak, which is a region of increased photoreceptor and ganglion cell density adapted for panoramic vision. This structure is not equivalent to the indented, specialized fovea found in primates. Consequently, the parafoveal region could not be assessed in our study. While our findings demonstrate neuroprotective effects in retinal layers vulnerable to silicone oil toxicity, including the GCL in the superior retina, future translational research should prioritize models that allow for macular assessment to confirm whether these drug combinations can preserve the GCL in the region most critical for human visual function. Second, the study focused primarily on histological changes and cell densities as indicators of neuroprotection. While these measures provide meaningful information, functional assessments such as ERG could not be included. Finally, the study did not directly explore the underlying mechanisms by which the drug combinations exert their neuroprotective effects. Further research elucidating the molecular pathways involved would enhance our understanding of the mechanisms of action and potentially identify additional targets for neuroprotection.

## Conclusions

The combination of ketamine, lamotrigine, and ketoprofen showed promising neuroprotective effects in a rabbit model following PPV and SOI, resulting in significant improvements in cell densities in the ONL, INL, and GCL compared with those in the saline-operated group and similar cell densities compared to negative controls. Notably, the combination groups exhibited greater levels of protection than the KTP alone group. These findings suggest the potential synergistic effects of these drug combinations in preserving retinal integrity. Further studies are needed to validate these results in human subjects and to explore the functional implications of the observed neuroprotective effects. Overall, our study contributes to the understanding of potential therapeutic strategies for neuroprotection in retinal pathologies and highlights the need for further research in this area.

## Data Availability

The datasets used and/or analyzed during the current study are available from the corresponding author on reasonable request.

## Abbreviations

PPV: Pars plana vitrectomy
IOP: Intraocular pressure
OPL: Outer plexiform layer
ROS: Reactive oxygen species
SOI: Silicone oil injection
INL: Inner nuclear layer
GCL: Ganglion cell layer
KTM: Ketamine
LMT: Lamotrigine
KTP: Ketoprofen
NSAID: Nonsteroidal anti-inflammatory drug
ANOVA: Analysis of variance
SEM: Standard error of the mean
TUNEL: Terminal deoxynucleotidyl Transferase dUTP Nick End Labeling
NMDA: N-methyl-D-aspartate
PBS: Phosphate-Buffered Saline
SSC: Saline-Sodium Citrate
ONL: Outer nuclear layer
IPL: Inner plexiform layer
MS: Multiple sclerosis
AMPK/mTOR: AMP-activated Protein Kinase/mammalian Target of Rapamycin
TFEB: Transcription Factor EB
